# Influence of Facility Size on Perioperative Outcomes in Minimally Invasive Esophagectomy for 14 152 Patients With Esophageal Cancer Based on the Japanese National Clinical Database: A Multicenter Cohort Study

**DOI:** 10.1002/ags3.70027

**Published:** 2025-05-01

**Authors:** Taro Oshikiri, Hisateru Tachimori, Hiroaki Miyata, Yoshihiro Kakeji, Ken Shirabe

**Affiliations:** ^1^ Department of Gastrointestinal Surgery and Surgical Oncology Ehime University Graduate School of Medicine Toon Ehime Japan; ^2^ Division of Gastrointestinal Surgery, Department of Surgery, Graduate School of Medicine Kobe University Kobe Hyogo Japan; ^3^ Database Committee The Japanese Society of Gastroenterological Surgery Tokyo Japan; ^4^ Endowed Course for Health System Innovation Keio University School of Medicine Tokyo Japan; ^5^ Department of Healthcare Quality Assessment, Graduate School of Medicine The University of Tokyo Tokyo Japan; ^6^ Department of Health Policy and Management Keio University School of Medicine Tokyo Japan; ^7^ The Japanese Society of Gastroenterological Surgery Tokyo Japan

**Keywords:** facility size, Japanese National Clinical Database, minimally invasive esophagectomy, mortality

## Abstract

**Background:**

Conventional minimally invasive esophagectomy (C‐MIE) is the mainstay for locally advanced esophageal cancer. However, the relationship among facility size, risk‐adjusted mortality and morbidity in C‐MIE remains unclear. This study aims to clarify whether C‐MIE should be consolidated into high‐volume centers in Japan.

**Methods:**

Risk models for perioperative mortality and morbidity were created using the Japanese National Clinical Database (NCD) data. NCD data registered between January 2016 and December 2020, including 14 152 C‐MIE records. The developed risk models were used to estimate the ratio of expected to observed events (perioperative deaths or complications) (O/E ratio) for each facility.

**Results:**

Regarding the risk model performances, the C‐indices of the perioperative mortality risk prediction models were 0.793. The O/E ratio and 95% confidence interval (CI) for perioperative mortality were facility size < 10 MIEs/year, O/E ratio: 1.368 and 95% CI: 1.140–1.597; facility size 10–29 MIEs/year, O/E ratio: 0.886 and 95% CI: 0.644–1.127; and facility size ≥ 30 MIEs/year, O/E ratio: 0. 61 and 95% CI: 0.342–0.892. Conversely, there were no significant differences in morbidity rate by facility size.

**Conclusions:**

The risk of perioperative mortality from C‐MIE was lower in hospitals with larger facilities than those with smaller facilities; therefore, consolidating patients for C‐MIE in high‐volume hospitals is necessary.

## Graphical abstract

This multicenter study investigated 14 152 minimally invasive esophagectomies (MIEs) using data from the Japanese National Clinical Database and showed a significantly lower operative mortality rate in high‐volume centers than in low‐volume centers. However, no differences in morbidity rates were found between facility sizes. MIEs should be consolidated into high‐volume centers.

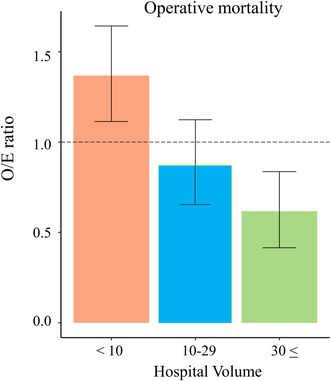

## 1 Introduction

Esophageal cancer is a common cancer, ranking seventh in incidence and cancer‐related deaths worldwide [1]. Esophagectomy with lymphadenectomy remains the mainstay for locally advanced esophageal cancer [2, 3]; however, the mortality and morbidity rates of open esophagectomy for this disease are high because of the extreme invasiveness [4, 5].

In 1992, Cuschieri was the first to report the so‐called conventional minimally invasive esophagectomy (MIE) (C‐MIE), which includes endoscopic esophagectomies under the prone or left lateral decubitus position through a right thoracoscopic approach [6]. Subsequently, some investigators have reported the utility of C‐MIE under the left lateral decubitus position [7, 8]. Furthermore, because it is less invasive, C‐MIE performed under the prone position spread rapidly worldwide, with the report of 130 cases by Palanivelu [9]. In particular, the reduction of respiratory complications, including pneumonia, which is associated with a worse overall prognosis [10], was one of the major factors in the rapid spread of C‐MIE. In contrast, the usefulness of upper mediastinal lymphadenectomy in C‐MIE has also been reported [11]. Furthermore, to reduce recurrent laryngeal nerve (RLN) paralysis, which is known to be a risk for pneumonia [12], some novel procedures for lymph node dissection around the RLN in C‐MIE have been reported [13, 14, 15]. As a result, C‐MIE has become more standardized and stable, which continues to this day.

Given the recognized importance of functional preservation, including thoracic duct preservation [16], and the need for further reduction of surgical invasiveness, case consolidation should be considered as the next step to stabilize MIE outcomes further. A previous study revealed that perioperative mortality rates after esophagectomy were low in high‐volume hospitals [17]. However, the differences in the risk of mortality and morbidity related to patients undergoing MIE in high‐ and low‐volume hospitals remain unclear.

The Japanese National Clinical Database (NCD) has registered various perioperative data since January 2011. Specifically, the number of cases registered with the NCD is approximately 95% of all surgical procedures performed in over 5000 hospitals in Japan; therefore, its data reflect the real‐world status [18].

Using the NCD data, we aimed to clarify the relationship between facility size and risk‐adjusted mortality and morbidity after MIE in Japan.

## 2 Methods

### 2.1 Data Collection

This multicenter cohort analysis examined patients treated with MIE for esophageal cancer in Japan. The NCD comprehensively collected perioperative outcomes of surgical cases in Japan. From January 2016 to December 2020, 20 816 MIEs for esophageal cancer were registered with the NCD. Therefore, this population was included in this study.

Some examinations, including computed tomography, positron emission tomography, and esophagogastroduodenoscopy, were used to diagnose all patients at each institution. The Union for International Cancer Control (UICC, 7th edition) tumor‐node‐metastasis cancer staging system was used to diagnose the oesophagal cancer stage [19]. The inclusion criteria were as follows: (1) thoracic esophageal primary tumor; (2) histologically diagnosed adenocarcinoma or esophageal squamous cell carcinoma; (3) thoracoscopic esophagectomy under the prone or left decubitus via right thoracic cavity; (4) clinical T (cT)1/2/3, clinical N (cN)0/1/2/3, and clinical M (cM)0/1 disease; (5) use of a gastric conduit as a reconstruction organ; and (6) cervical anastomosis (McKeown esophagectomy). Patients who had undergone robot‐assisted surgery, which was approved in 2018 in the Japanese insurance system, were excluded because of the disadvantages of mixing unstable data from the introduction period with C‐MIE. Staged reconstructions were also excluded. Cases where the patients refused participation or records were missing were excluded.

### 2.2 Validation of NCD Dataset

Standardized data entry: A uniform format is used across participating facilities nationwide to ensure consistency in data entry.

Multi‐step quality checks: Data are checked at multiple stages during entry, registration, and analysis to detect errors and inconsistencies.

External audits: External audits are conducted by experts at randomly selected centers to check registration data against source documents to ensure accuracy.

Of the 14 152 cases that met the selection criteria for this study, 13 676 in the mortality analysis and 13 677 in the morbidity analysis were free of missing variables for multivariable analysis, for a missing rate of < 4%.

### 2.3 Endpoints

The primary endpoint was perioperative mortality, which was defined as death during hospitalization or 30‐day mortality. In contrast, the secondary endpoint was comprehensive perioperative morbidity greater than grade III of the Clavien–Dindo (C–D) classification [20]. Morbidity includes anastomotic leakage, pneumonia, recurrent laryngeal nerve palsy, atelectasis, chylothorax, unplanned intubation, prolonged mechanical ventilation over 48 h, need for transfusion, deep vein thrombosis, sepsis, heart failure, and gastric conduit necrosis. Each of them was defined as Appendix S1.

The Ethics Committee of Kobe University and the Japanese Society of Gastroenterological Surgery approved the study protocol (approval number: 20190128). All hospitals participating in the NCD project were given the option to opt out. The Ethics Committee of the NCD approved the retrospective analyses of the NCD data for observational research.

### 2.4 Development of the Risk Model for Perioperative Mortality and Morbidity

Risk models for perioperative mortality and morbidity were created based on the NCD data registered from January 2016 to December 2020, which were extracted from 14 152 records.

First, frequency distribution tables of the outcomes were made. Next, cross‐tables based on outcomes and candidate risk factors were created. The covariates believed to be risk factors were sex, age, body mass index (BMI), weight loss < 10% within 6 months preoperatively, preoperative state of activities of daily living (ADL), American Society of Anesthesiologists physical status classification (ASA‐PS), Brinkman index, alcohol consumption, diabetes mellitus, chronic steroid use, anticoagulant therapy, hypertension, angina pectoris, congestive heart failure, chronic obstructive pulmonary disease (COPD), preoperative dialysis, previous cerebrovascular disease, preoperative chemotherapy and/or radiotherapy, cT and cN factors according to the classification of the UICC 7th edition [19], white blood cell, hemoglobin, albumin, creatinine, C‐reactive protein (CRP), year of surgery, position at thoracoscopic surgery, and reconstruction route.

Each risk model was created by a forward–backward stepwise logistic regression using Akaike Information Criterion (AIC). The risk model minimizing the AIC for predicting the event of each outcome was selected. The step AIC function from the MASS package (R) was used for this procedure. The assessed odds ratios (ORs) and 95% confidence intervals (CIs) for each coefficient were calculated. In addition, the receiver operating characteristic (ROC) curve and the area under the ROC curve (AUC) were used to assess the discriminative performance of each model. The C‐index, equivalent to the AUC, is a measure of goodness of fit for logistic regression. All tests were two‐tailed, and *p* < 0.05 was considered statistically significant. R software version 4.3 or later (R Foundation, Vienna, Austria) was used to perform each statistical analysis.

### 2.5 Calculation of Facility Size

Facility size was categorized based on the mean number of MIEs per year. Based on the previous report [17], hospitals were classified into the following three groups: hospitals that treated < 10, 10–29, and ≥ 30 cases.

### 2.6 Estimation of the Adjusted Mortality and Morbidity by Facility Size

The risk models assessed the expected number of events (perioperative deaths or complications) in each stratum by summing the predicted probabilities for each facility size stratum. In addition, the ratio of expected to observed events (perioperative deaths or complications) (O/E ratio) and 95% CI were calculated using the bootstrap method. The count of bootstrap iterations was set to 5000.

## 3 Results

### 3.1 Risk Profile for Mortality and Morbidity in the Study Population

**FIGURE 1:**
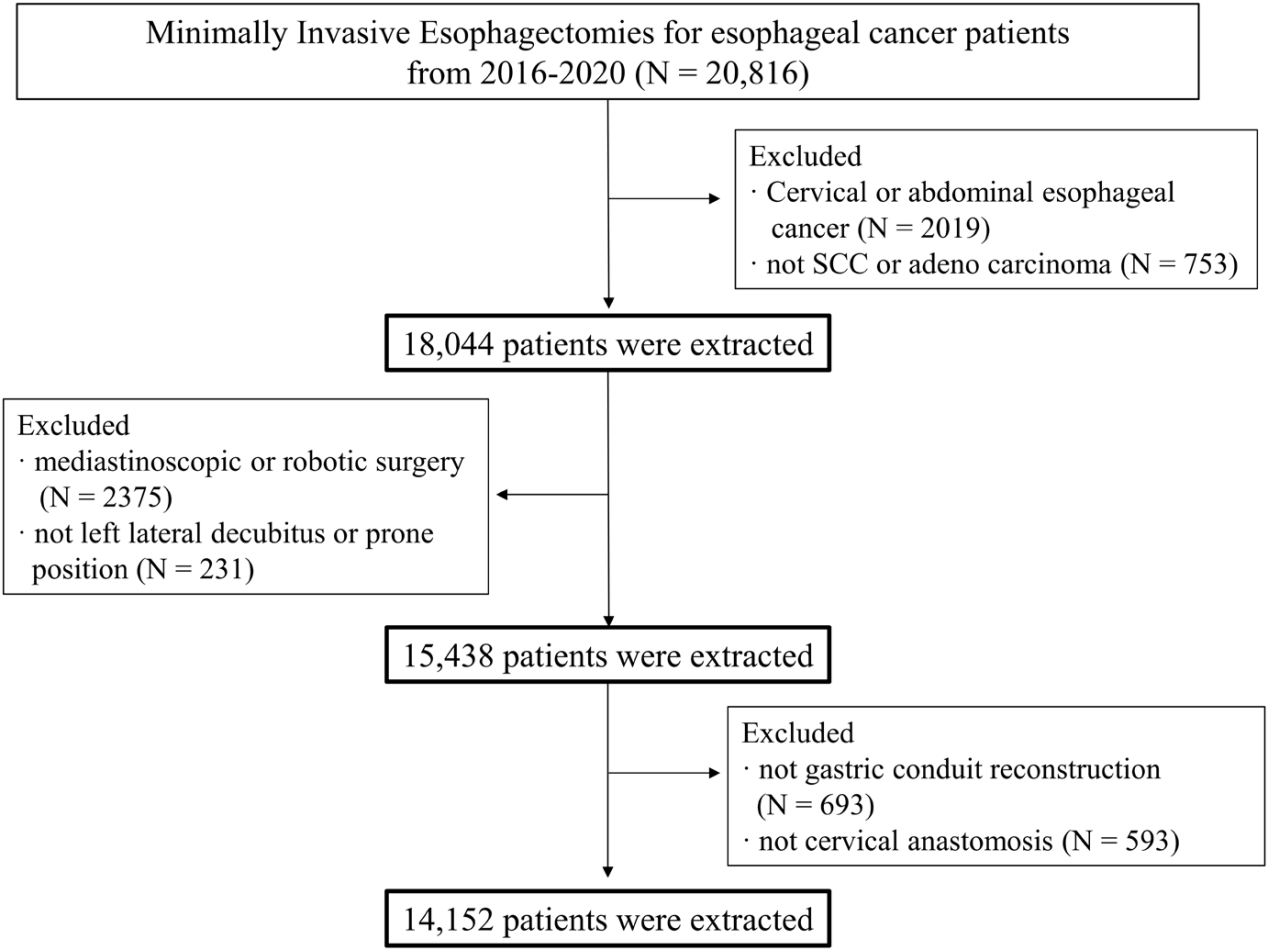
Flowchart of patient enrolment. Among the patients who participated in the Japanese National Clinical Database (NCD) from 2016 to 2020, 14 152 minimally invasive esophagectomies (MIEs) in 566 hospitals were included in this study.

Among the patients enrolled in the NCD between January 2016 and December 2020, 14 152 treated with MIE in 566 hospitals participated in this study (Figure 1). Table, Appendix S2 shows the patient characteristics and the associated risk profile. The perioperative mortality rate in MIE was 1.4% for this study. In detail, a mortality rate of > 3.0% was observed in patients aged ≥ 81 years (4.7%) and those with low ADL (in any assistance; 3.5%, in full assistance; 19%), ASA‐PS classification of 3 or more (4.4%), chronic steroid use (3.9%), history of angina pectoris within 30 days (3.1%), and history of cerebrovascular disease (3.3). Morbidities with C–D classification ≥ grade III were observed in 21% of patients. In detail, a morbidity (C–D classification ≥ grade III) rate of > 25% was observed in patients with weight loss (> 10%), low ADL (in any assistance; 27%, in full assistance; 25%), ASA‐PS classification of 3 or more (27%), chronic steroid use (33%), anticoagulant therapy (25%), and COPD (29%) (see Table, Appendix S2, characteristics of the study cohort).

### 3.2 Risk Model and Performance for Perioperative Mortality and Morbidity

Variables and risk factors for each outcome were selected using the AIC (Tables 1 and 2). Specifically, variables were selected for each risk model for each outcome to differ from the risk factors chosen for each risk model. Multivariate logistic regression analysis of perioperative mortality revealed that age, sex, BMI, ADL, ASA‐PS classification, alcohol consumption, diabetes mellitus, chemotherapy, radiotherapy, cN, albumin, CRP, and position at the time of MIE were significant risk factors (Table 1). In the morbidity analysis, sex, BMI, ASA‐PS classification, Brinkman index, diabetes mellitus, chronic steroid use, COPD, dialysis, previous cerebrovascular disease, radiotherapy, serum creatinine, CRP, procedure year, position at the time of MIE, and reconstruction route were significant risk factors (Table 2).

**TABLE 1:** Risk models for predicting perioperative mortality in MIE.

Variables	Multivariate analyses		
	OR	95% CI	*p*
Age			
Under 50 years	—	—	—
51–70 years	2.59	0.56–46.1	0.3
71–80 years	5.55	1.21–98.7	0.091
81 and over years	12.6	2.63–226	0.013
Sex			
Female	—	—	—
Male	2.02	1.27–3.35	0.004
BMI			
< 18.5 kg/m^2^	—	—	—
≥ 18.5, and < 25 kg/m^2^	0.63	0.45–0.91	0.012
≥ 25 kg/m^2^	0.41	0.21–0.74	0.004
ADL within 30 days			
Independence	—	—	—
Any assistance	0.91	0.27–2.27	0.9
Full assistance	19.8	4.14–71.7	< 0.001
ASA classifications			
1	—	—	—
2	1.88	0.97–4.23	0.087
3, 4 and 5	5.26	2.58–12.2	< 0.001
Alcohol consumption			
No	—	—	—
Occasional drinking	0.83	0.49–1.36	0.5
Habitual drinking	0.57	0.40–0.81	0.002
Diabetes			
No	—	—	—
Yes	1.31	0.91–1.87	0.14
Cancer chemotherapy within 30 days			
−	—	—	—
+	0.68	0.45–1.01	0.063
Cancer radiotherapy within 90 days			
−	—	—	—
+	1.65	0.89–2.82	0.084
cN			
cN0	—	—	—
cN1‐3	1.28	0.94–1.75	0.13
Albumin			
≥ 4.0 g/dL	—	—	—
< 4.0 g/dL	1.78	1.27–2.1	< 0.001
CRP			
≤ 0.1 mg/dL	—	—	—
> 0.1 mg/dL	1.63	1.19–2.25	0.003
Position at thoracoscopic surgery			
Left lateral decubitus	—	—	—
Prone	0.55	0.41–0.76	< 0.001

**TABLE 2:** Risk models for predicting perioperative morbidity ≥ grade III in MIE.

Variables	Multivariate analyses		
	OR	95% CI	*p*
Sex			
Female	—	—	—
Male	1.14	1.01–1.28	0.036
BMI			
< 18.5 kg/m^2^	—	—	—
≥ 18.5, and < 25 kg/m^2^	0.91	0.81–1.01	0.080
≥ 25 kg/m^2^	1.03	0.89–1.21	0.7
ASA classifications			
1	—	—	—
2	1.19	1.04–1.35	0.010
3, 4 and 5	1.42	1.18–1.70	< 0.001
Brinkman index			
< 200	—	—	—
≥ 200, and < 400	1.12	0.96–1.32	0.2
≥ 400	1.21	1.09–1.34	< 0.001
Diabetes			
No	—	—	—
Yes	1.11	0.99–1.25	0.072
Chronic steroid use			
No	—	—	—
Yes	1.53	1.04–2.23	0.028
COPD			
No	—	—	—
Yes	1.46	1.27–1.67	< 0.001
Dialysis within 14 days			
No	—	—	—
Yes	2.43	1.22–4.84	0.011
Previous cerebrovascular disease			
No	—	—	—
Yes	1.20	0.96–1.48	0.10
Cancer radiotherapy within 90 days			
−	—	—	—
+	1.16	0.95–1.39	0.14
Serum creatinine			
≤ 1.04 (men) or 0.79 (women) mg/dL	—	—	—
> 1.04 (men) or 0.79 (women) mg/dL	1.10	1.00–1.21	0.052
CRP			
≤ 0.1 mg/dL	—	—	—
> 0.1 mg/dL	1.18	1.08–1.28	< 0.001
Year			
2016	—	—	—
2017	1.18	1.02–1.36	0.025
2018	1.16	1.01–1.33	0.038
2019	1.02	0.89–1.18	0.8
2020	1.16	1.01–1.34	0.039
Position at thoracoscopic surgery			
Left lateral decubitus	—	—	—
Prone	0.93	0.84–1.02	0.11
Reconstruction route			
Posterior mediastinal	—	—	—
Presternal	1.91	1.49–2.42	< 0.001
Retrosternal	1.25	1.14–1.37	< 0.001

**FIGURE 2:**
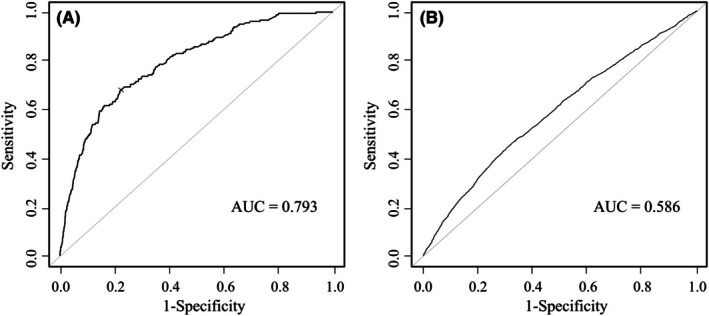
Receiver operating characteristic curves of mortality and morbidity. The C‐indices were (A) 0.793 for operative mortality and (B) 0.586 for morbidity ≥ grade III.

The C‐index, expressed as an AUC, and model calibration were evaluated across the entire risk group to assess the risk model's performance. The C‐indices of the perioperative mortality and morbidity (C‐D classification ≥ grade III) risk prediction models were 0.793 (Figure 2A) and 0.586 (Figure 2B), respectively.

### 3.3 Distribution of Facility Size

**FIGURE 3:**
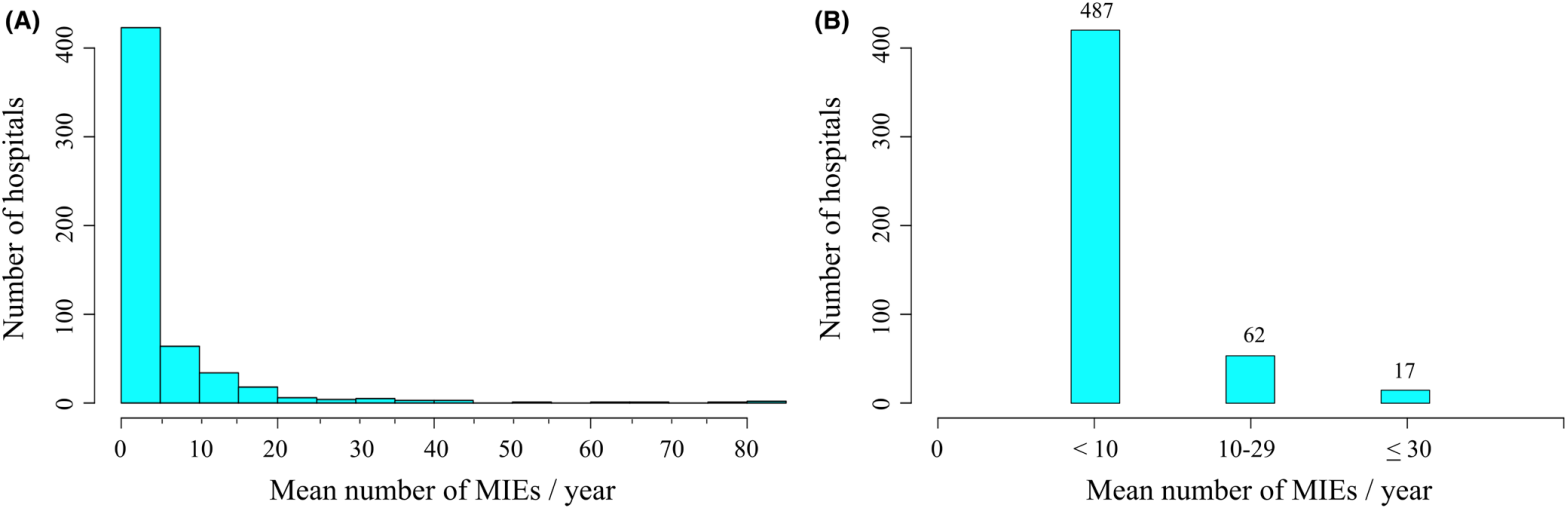
Number of hospitals in detail. (A) A total of 487, 52, 10, 8, and 9 hospitals treated < 10, 10–19, 20–29, 30–39, and > 40 cases per year, respectively. (B) The number of hospitals was classified into the following three groups: 487, 62, and 17 hospitals treated < 10, 10–29, and ≥ 30 cases per year, respectively.

In total, 487 hospitals treated < 10 cases annually. In addition, 52, 10, 8, and 9 hospitals treated 10–19, 20–29, 30–39, and ≥ 40 cases per year, respectively (Figure 3A). Next, based on the above data, these hospitals were categorized into the following three groups: 487, 62, and 17 hospitals treated < 10, 10–29, and ≥ 30 cases per year, respectively (Figure 3B). In each group, the number of patients was 5198, 4827, and 4127, respectively.

### 3.4 Risk‐Adjusted Operative Mortality by Facility Size

**FIGURE 4:**
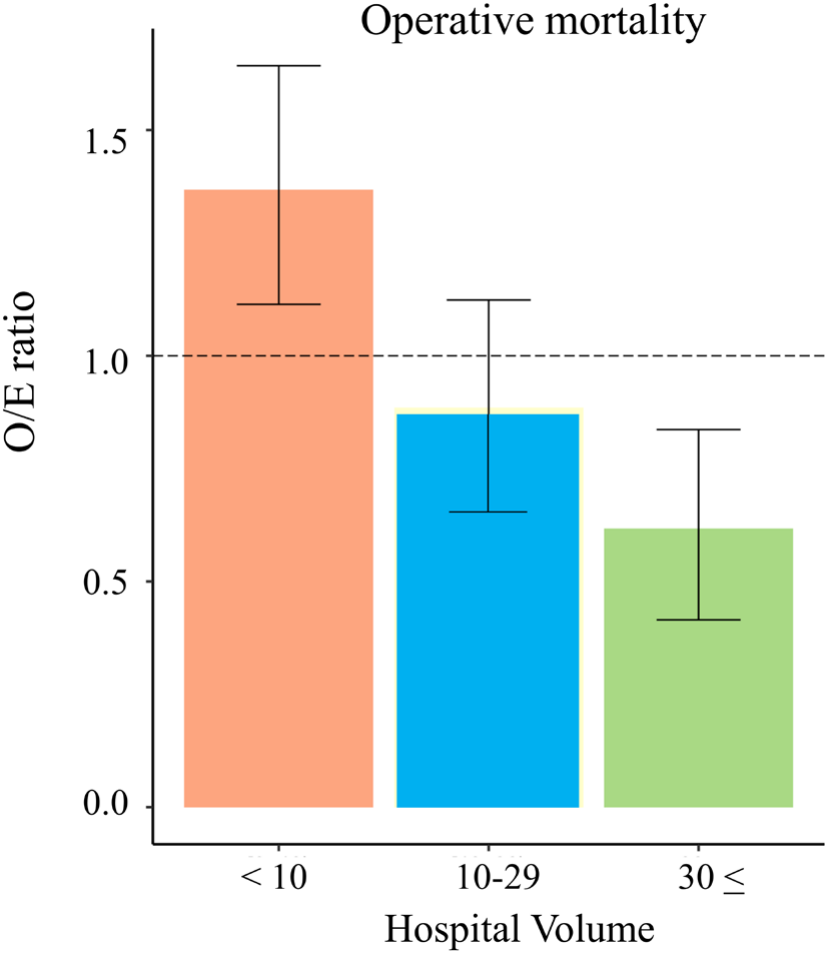
The O/E ratio for perioperative mortality for facility sizes. The ratios of expected to observed events (O/E ratio) for perioperative mortality for facility sizes of < 10, 10–29, and ≥ 30 were 1.368, 0.886, and 0.617, respectively.

The facility size was categorized into three groups (facility size < 10, 10–29, and ≥ 30). Mortality rates of whole and each group were 1.3%, 1.8%, 1.2%, and 0.7%, respectively (Table 3) The O/E ratio and 95% CI for perioperative mortality were as follows: facility size < 10; O/E ratio: 1.368 and 95% CI: 1.140–1.597; facility size 10–29, O/E ratio: 0.886 and 95% CI: 0.644–1.127; and facility size ≥ 30, O/E ratio: 0.617 and 95% CI: 0.342–0.892 (Table 4, Figure 4A).

**TABLE 3:** Perioperative mortality and morbidity by hospital volume.

Variables	Whole cohort	Hospital volume		
		< 10	10–29	30 ≤
	Patient number			
	*N* = 14 152	*N* = 5198	*N* = 4827	*N* = 4127
Anastomotic leakage	2007 (14%)	839 (16%)	699 (14%)	469 (11%)
Pneumonia	2008 (14%)	762 (15%)	663 (14%)	583 (14%)
Recurrent laryngeal nerve palsy	2151 (15%)	834 (16%)	764 (16%)	553 (13%)
Atelectasis	661 (4.7%)	281 (5.4%)	222 (4.6%)	158 (3.8%)
Chylothorax				
Unplanned intubation	624 (4.4%)	305 (5.9%)	177 (3.7%)	142 (3.4%)
Prolonged mechanical ventilation over 48 h	719 (5.1%)	411 (7.9%)	177 (3.7%)	131 (3.2%)
Need for transfusion	932 (6.6%)	408 (7.8%)	264 (5.5%)	260 (6.3%)
Deep vein thrombosis	167 (1.2%)	48 (0.9%)	73 (1.5%)	46 (1.1%)
Sepsis	55 (0.4%)	29 (0.6%)	15 (0.3%)	11 (0.3%)
Heart failure	51 (0.4%)	27 (0.5%)	16 (0.3%)	8 (0.2%)
Gastric conduit necrosis	71 (0.5%)	33 (0.6%)	27 (0.6%)	11 (0.3%)
Mortality	182 (1.3%)	96 (1.8%)	56 (1.2%)	30 (0.7%)

**TABLE 4:** O/E ratio and CI of operative mortality and morbidity by hospital volume.

Hospital volume	Operative mortality				Morbidity			
	Expected number	Observed number	OE ratio	95% CI	Expected number	Observed number	OE ratio	95% CI
< 10	70	96	1.368	1.140–1.597	1039	1114	1.073	1.019–1.126
10–29	63	56	0.886	0.644–1.127	994	950	0.956	0.901–1.010
≤ 30	49	30	0.617	0.342–0.892	894	863	0.965	0.908–1.023

Abbreviations: CI, 95% confidence interval; O/E, ratio of the expected number to the observed number.

### 3.5 Risk‐Adjusted Operative Morbidity by Facility Size

**FIGURE 5:**
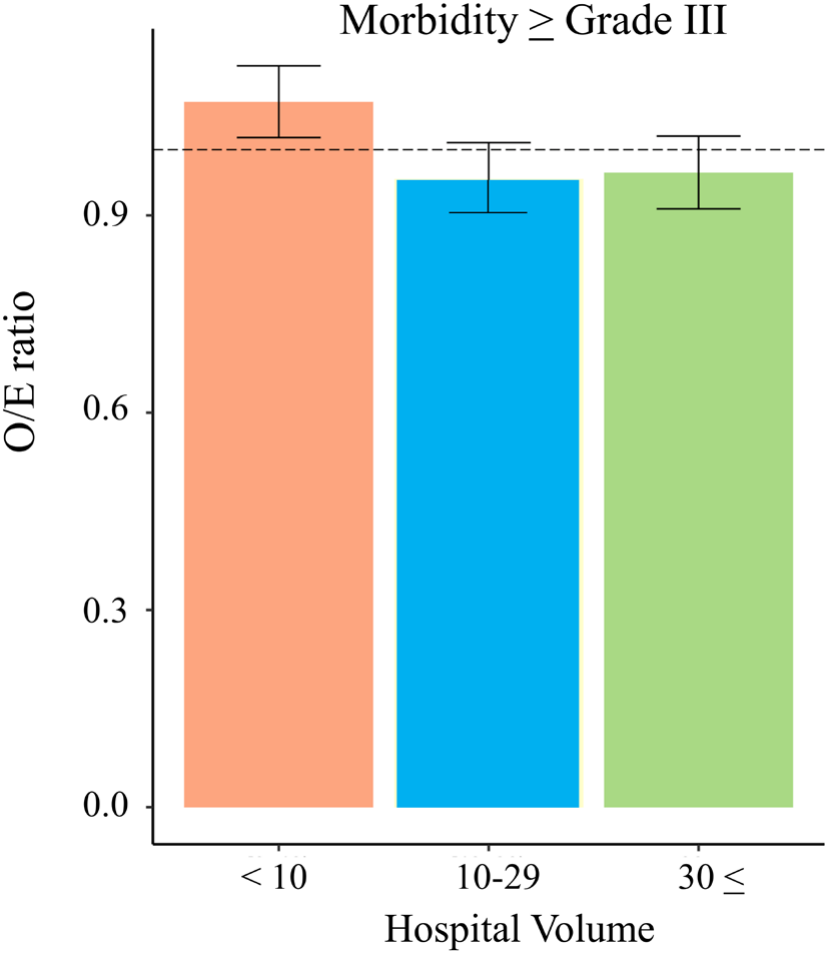
The O/E ratio for perioperative morbidity for facility sizes. The ratios of expected to observed events (O/E ratio) for morbidity ≥ grade III for facility sizes of < 10, 10–29, and ≥ 30 were 1.073, 0.956, and 0.965, respectively.

The facility size was classified into three groups, and the number of complications in each of the three groups is shown in Table 3. The O/E ratio and 95% CI for morbidity were as follows: facility size < 10; O/E ratio: 1.073 and 95% CI: 1.019–1.126; facility size 10–29, O/E ratio: 0.956; 95% and CI: 0.901–1.010; and facility size ≥ 30, O/E ratio: 0.965 and 95% CI: 0.908–1.023 (Table 4, Figure 5A).

## 4 Discussion

In this study, we showed that the risk of mortality during MIE for esophageal cancer is lower in high‐volume hospitals that treat ≥ 30 cases of MIE than in low‐volume hospitals that treat < 10 cases of MIE per year. The O/E ratios were 0.617 and 1.368, respectively. In the subgroup analysis (data not shown), which was divided into groups of 10 cases each, the O/E ratio for mortality tended to be less than 1 even in facilities with 20 to 29 cases per year. Since detailed classification may lead to discrepancies in some groups, we have divided the data into three major groups, but the minimum annual volume of C‐MIE may be 20 or more cases per year. However, the O/E ratio of comprehensive morbidity rates higher than grade III was approximately 1.0, regardless of the facility size. In hospitals with < 10 cases per year, the O/E ratio was the worst, at 1.073. These results indicate that morbidity did not differ significantly by facility size, whereas mortality varied greatly. Assuming that differences in patient background between facilities are minimal because of risk adjustment, morbidity occurs with a certain frequency regardless of the facility size; however, high‐volume centers can better manage perioperative problems than low‐volume centers, resulting in lower mortality rates. Supporting previous reports showed that esophagectomy outcomes in the esophagectomy‐targeted hospital were superior to those in standard ACS‐NSQIP hospitals. In this study, 5449 (71%) patients underwent esophagectomy at esophagectomy‐targeted centers, while 2181 (29%) patients underwent esophagectomy at standard centers. Mortality rate was significantly lower in esophagectomy‐targeted hospitals (2% vs. 4%, *p* < 0.01) even though there was no difference in severe morbidity (21% vs. 23%, *p* = 0.09). This reflects the observed difference in failure‐to‐rescue (defined as mortality after the development of severe morbidity) (11% vs. 17%, *p* < 0.01), which indicates that targeted centers may be superior in managing postoperative complications after esophagectomy [21]. Our results are very consistent with these ACS‐NASQIP data. Of the 566 hospitals, the number of facilities that treated ≥ 30 MIEs per year was only 17. Therefore, further consolidation of patients in specific facilities is needed in the future.

In this study, facility sizes were compared using risk‐adjusted models. Constructing accurate risk adjustment models is important. Consequently, the C‐index of the perioperative mortality risk prediction model in this study was 0.793. Compared to previous reports on risk models using the NCD data, that of esophagectomy performed in 2011 reported by Takeuchi et al. was 0.742 [22]. In addition, the latest risk models of esophagectomy derived from 32 779 cases registered with the NCD between 2012 and 2017, showed that the C‐indices of the perioperative mortality risk prediction model was 0.721 [23]. Compared to the previous data, the risk model established in this study appears to have excellent prediction accuracy and reliability. Therefore, the results of the primary endpoint of this study based on such a superior risk‐adjusted model are considered reliable. Also, not only size but also quality is important in the discussion about the facility. Previous reports showed that institutions accredited by the Japan Esophageal Society as specialized centers for esophagectomy show better short‐ and long‐term outcomes than non‐accredited institutions [24, 25]. Regarding morbidity, the accuracy of the risk‐adjusted model was somewhat reduced, with a C‐index of 0.586. Several reasons exist for this finding. Surgical techniques that lead to complications involve complicated factors, including preoperative patient status. Despite the diversity of the complications, the analysis of only the comprehensive complication in this study may have also resulted in less accurate predictions. Additionally, one of the reasons for this is that the registration system does not reflect the severity of each complication in detail, only the presence or absence of it. Brushing up on a registration system based not only on the presence or absence of morbidities, but also on grade, is expected to improve the model's performance. Incidentally, we recently reported a highly accurate risk‐adjusted model created using the same NCD data and methodology; therefore, test data were not used to validate the model this time [23].

Previous studies have reported low mortality after esophagectomy in high‐volume centers [26, 27, 28]; however, these are all systematic reviews and not risk‐model‐based analyses of big data. One Japanese study adjusted for risk factors associated with mortality and evaluated the facility size–outcome relationship after esophagectomy. In that study, 16 556 patients treated with esophagectomy at 988 hospitals in Japan between 2011 and 2013 were extracted from the NCD database. They concluded that risk‐adjusted operative mortality rates after esophagectomy were lower in large hospitals than in small hospitals [17]. However, one limitation of that study is that it included all cases where esophagectomies were performed rather than only cancer cases. Moreover, the percentage of MIEs performed was only 31.0% in 2011, 37.0% in 2012, and 40.7% in 2013. In addition, C‐MIE was in its introductory phase at that time, and its performance was unstable [22]. Our study is novel from two strict points of view: (1) it is limited to C‐MIE via the right thoracic cavity performed only for esophageal cancer, and (2) 3–8 years have passed since the previous study, and C‐MIE procedures have become standardized and stable in Japan. Of course, the possibility that a trainee has done C‐MIE should be considered, and may be a confounding factor. However, as previously reported [17], we believe that outcomes of C‐MIE also depend on the experience of the institution, not on the surgeon's excessive experience. As for Robot‐assisted MIE (RAMIE), which was introduced in 2018 as a Japanese insurance treatment, due to the limitation of the number of surgical Robots in each hospital, even high‐volume centers probably account for only about half of the total number of esophagectomies. Consequently, we assume that this will not have a significant impact on the size of C‐MIE facilities, especially until 2020.

Our conclusions recommend centralization of C‐MIE to high‐volume centers. While the data support this notion, practical considerations such as patient accessibility, healthcare infrastructure, and resource allocation should also be acknowledged. In fact, in remote areas, there are problems such as difficulties in getting to a remote high‐volume center, providing care by family members, and so on, so it is necessary to consider a variety of balances.

This study had some limitations. First, the risk model was based only on the preoperative factors. If a more accurate morbidity prediction model that includes intraoperative and other factors is used, there could be differences in morbidities based on facility size. However, intraoperative factors, such as blood loss and operative time, may already be influenced by facility size, which we consider to be controversial. Second, because the actual situation, perioperative management, and results of esophagectomy, including C‐MIE, vary considerably across countries, the results of this study cannot be directly extrapolated to other countries. However, in conjunction with previous reports from systematic reviews, similar trends will likely be observed for C‐MIE. Third, RAMIEs were excluded from this study. It is highly likely that facilities where robotic surgery is the mainstream are high‐volume centers that perform more than 30 MIEs per year, and it is assumed that C‐MIE was the mainstream in such high‐volume centers until 2018. Therefore, the results of this analysis can be extrapolated to those centers, and may help in the decision to have trainees do C‐MIE before starting robotic surgery. Moreover, future analyses similar to this study limited to RAMIE are needed.

## 5 Conclusions

In the strictly limited population of patients with C‐MIE for esophageal cancer, a disparity was observed in facility size based on a risk‐adjusted mortality model. Consequently, the risk of mortality from C‐MIE in Japan is lower in hospitals with larger facilities than in those with smaller facilities. Therefore, consolidating patients in high‐volume hospitals will be necessary in the future.

## Author Contributions

**Taro Oshikiri:** investigation, writing – original draft, conceptualization, visualization, writing – review and editing, project administration. **Hisateru Tachimori:** methodology, software, data curation, validation. **Hiroaki Miyata:** methodology, data curation, formal analysis, resources. **Yoshihiro Kakeji:** conceptualization, formal analysis. **Ken Shirabe:** supervision.

## Ethics Statement

Approval of the research protocol: The Ethics Committee of Kobe University and the Japanese Society of Gastroenterological Surgery approved the study protocol (approval number: 20190128). The Ethics Committee of the NCD approved the retrospective analyses of the NCD data for observational research.

## Consent

All hospitals participating in the NCD project were given the option to opt‐out.

## Conflicts of Interest

Hiroaki Miyata is affiliated with the Department of Health Care Quality Assessment, Graduate School of Medicine, University of Tokyo. The department is a social collaboration with grants from the National Clinical Database, Johnson & Johnson K.K., Nipro Corporation, and Intuitive Surgical Sàrl. Hisateru Tachimori holds an endowed chair at Keio University, funded by Takeda Pharmaceutical Company Limited, and the Department of Healthcare Quality Assessment at the University of Tokyo with grants from the National Clinical Database, Johnson & Johnson K.K., Nipro Corporation, and Intuitive Surgical Sàrl. All authors, including Dr. Miyata and Dr. Tachimori, have no conflicts of interest to declare. This study was supported by a grant from the Japanese Society of Gastroenterological Surgery (grant number: APJ‐2019‐02). Dr. Yoshihiro Kakeji and Dr. Ken Shirabe are the Editorial Board Member of AGS.

## Supporting information


Appendix S1.

Appendix S2.
