# Supplementary material for: Influence of Facility Size on Perioperative Outcomes in Minimally Invasive Esophagectomy for 14 152 Patients With Esophageal Cancer Based on the Japanese National Clinical Database: A Multicenter Cohort Study
Source: Ann Gastroenterol Surg. 2025 May 1;9(5):942–51. doi: 10.1002/ags3.70027 (PMC12414597; doi:10.1002/ags3.70027)
Supplement: Supplementary file 1 — Appendix S1. [file AGS3-9-942-s001.docx]

★Supplemental Digital Content 1

Definition of each complication

| Anastomotic leakage | If drainage is present or drainage is required, or if no drainage is present but is documented by imaging |
| --- | --- |
| Pneumonia | Pneumonia on chest x-ray or CT, or positive sputum bacterial culture |
| Recurrent laryngeal nerve palsy | Vocal cord failure due to recurrent nerve paralysis |
| Atelectasis | Collapsed lung parenchyma on imaging |
| Chylothorax | Pleural effusion of more than 1000 ml/day, sometimes cloudy |
| Unplanned intubation | Unscheduled reintubation after being extubated within 30 days postoperatively due to respiratory or cardiac failure |
| Prolonged mechanical ventilation over 48 h | Condition under ventilator control for more than 48 hours |
| Need for transfusion | More than 1 unit of concentrated red blood cell or whole blood transfusion within 72 hours after returning from the operating room |
| Deep vain thrombosis | New deep vein thrombus or embolus that develops within 30 postoperative days and is associated with inflammation. Diagnosis is made by doppler, CT, and venography. |
| Sepsis | Life-threatening organ damage due to uncontrolled host response to infection |
| Heart failure | Decreased cardiac output |
| Gastric conduit necrosis | Necrosis of gastric conduit due to ischemia |
