# Supplementary material for: Influence of Facility Size on Perioperative Outcomes in Minimally Invasive Esophagectomy for 14 152 Patients With Esophageal Cancer Based on the Japanese National Clinical Database: A Multicenter Cohort Study
Source: Ann Gastroenterol Surg. 2025 May 1;9(5):942–51. doi: 10.1002/ags3.70027 (PMC12414597; doi:10.1002/ags3.70027)
Supplement: Supplementary file 2 — Appendix S2. [file AGS3-9-942-s002.docx]

★Supplemental Digital Content 2

Characteristics of the study cohort are summarized

|  | Entire study　population | Surgical outcomes | |
| --- | --- | --- | --- |
| variables |  | perioperative mortality | Morbidity > Grade III |
| Number of patients (% of the entire population) | n= 14152 | n=194 (1.4) | n=3009 (21) |
| **Demographics** |  |  |  |
| Age, Under 50 years | 502 | 1 (0.2) | 89 (18) |
| 51-70 years | 7318 | 56 (0.8) | 1497 (20) |
| 71-80 years | 5566 | 101 (1.8) | 1245 (22) |
| 81 and over years | 766 | 36 (4.7) | 178 (23) |
| Sex; male | 11430 | 170 (1.5) | 2516 (22) |
| female | 2722 | 24 (0.9) | 493 (18) |
| BMI; <18.5 Kg/㎡ | 2524 | 54 (2.1) | 555 (22) |
| ≥18.5, and <25 Kg/㎡ | 9923 | 125 (1.3) | 2054 (21) |
| ≥25 Kg/㎡ | 1705 | 15 (0.9) | 400 (23) |
| Weight loss, >10%; no | 13419 | 171 (2.3) | 2825 (21) |
| yes | 733 | 23 (3.1) | 184 (25) |
| **General** |  |  |  |
| ADL within 30 days; independence | 13989 | 186 (1.3) | 2965 (21) |
| any assistance | 147 | 5 (3.4) | 40 (27) |
| full assistance | 16 | 3 (19) | 4 (25) |
| ASA classifications; 3, 4 and 5 | 1206 | 53 (4.4) | 324 (27) |
| Brinkman index; <200 | 3822 | 58 (1.5) | 698 (18) |
| ≥200, and <400 | 1378 | 13 (0.9) | 275 (20) |
| ≥400 | 8952 | 123 (1.4) | 2036 (23) |
| Alcohol consumption; no | 2750 | 61 (2.2) | 549 (20) |
| occasional drinking | 1562 | 24 (1.5) | 344 (22) |
| habitual drinking | 9840 | 109 (1.1) | 2116 (22) |
| Diabetes; no | 12111 | 148 (1.2) | 2512 (21) |
| yes | 2041 | 46 (2.3) | 497 (24) |
| Chronic steroid use; no | 14023 | 189 (1.3) | 2967 (21) |
| yes | 129 | 5 (3.9) | 42 (33) |
| Anticoagulant therapy; no | 13150 | 170 (1.3) | 2763 (21) |
| yes | 1002 | 24 (2.4) | 246 (25) |
| **Cardiovascular factors** |  |  |  |
| Hypertension; no | 8276 | 96 (1.2) | 1671 (20) |
| yes | 5876 | 98 (1.7) | 1338 (23) |
| Angina pectoris within 30 days; no | 14054 | 191 (1.4) | 2990 (21) |
| yes | 98 | 3 (3.1) | 19 (19) |
| **Respiratory factors** |  |  |  |
| COPD; no | 13009 | 170 (1.3) | 2674 (21) |
| yes | 1143 | 24 (2.1) | 335 (29) |
| **Renal factors** |  |  |  |
| Dialysis within 14 days; no | 14115 | 193 (1.4) | 2991 (21) |
| yes | 37 | 1 (2.7) | 18 (49) |
| **Cerebral nerve factors** |  |  |  |
| Previous cerebrovascular disease; no | 13666 | 178 (1.3) | 2880 (21) |
| yes | 486 | 16 (3.3) | 129 (27) |
| **Oncological factors** |  |  |  |
| Cancer chemotherapy within 30 days; - | 11178 | 159 (1.4) | 2365 (21) |
| + | 2974 | 35 (1.2) | 644 (22) |
| Cancer radiotherapy within 90 days; - | 13509 | 179 (1.3) | 2849 (21) |
| + | 643 | 15 (2.3) | 160 (25) |
| cT; cT0-2 | 8152 | 80 (1.0) | 1697 (21) |
| cT3-4 | 5961 | 113 (1.9) | 1302 (22) |
| cN; cN0 | 6745 | 72 (1.1) | 1408 (21) |
| cN1-3 | 7379 | 118 (1.6) | 1594 (22) |
| **Preoperative laboratory data** |  |  |  |
| WBC; < 3500/mL or >9500/ml | 2069 | 43 (2.1) | 444 (21) |
| Hemoglobin; < 13.5 (men) or 11.5 (women) g/dl | 8765 | 143 (1.6) | 1920 (22) |
| Albumin; < 4.0 g/dl | 6155 | 133 (2.2) | 1390 (23) |
| Serum creatinine; >1.04 (men) or 0.79 (women) mg/dL | 3271 | 64 (2.0) | 764 (23) |
| CRP; > 0.1 mg/dL | 5189 | 111 (2.1) | 1249 (24) |
| **Procedure** |  |  |  |
| Year; 2016 | 2243 | 27 (1.2) | 422 (19) |
| 2017 | 2608 | 38 (1.5) | 569 (22) |
| 2018 | 3066 | 40 (1.3) | 679 (22) |
| 2019 | 3181 | 50 (1.6) | 649 (20) |
| 2020 | 3054 | 39 (1.3) | 690 (23) |
| Position at thoracoscopic surgery; prone | 10430 | 122 (1.2) | 2180 (21) |
| left lateral decubitus | 3722 | 72 (1.9) | 829 (22) |
| Reconstruction route; posterior mediastinal | 4539 | 62 (1.4) | 834 (18) |
| presternal | 362 | 5 (1.4) | 119 (33) |
| retrosternal | 9251 | 127 (1.4) | 2056 (22) |
